# Can HIPEC be used against platinum-resistance and for inducing sensitivity to PARP inhibitors in ovarian cancer?

**DOI:** 10.20517/cdr.2020.27

**Published:** 2020-07-10

**Authors:** Thales Paulo Batista, Graziela Zibetti Dal Molin

**Affiliations:** ^1^Department of Surgery/Oncology, IMIP - Instituto de Medicina Integral Professor Fernando Figueira, Recife 50070-550, PE, Brazil.; ^2^Department of Surgery, UFPE - Universidade Federal de Pernambuco, Recife 50070-902, PE, Brazil.; ^3^Department of Medical Oncology, Beneficência Portuguesa de São Paulo, São Paulo 01323-001, SP, Brazil.

**Keywords:** Injections, intraperitoneal, hyperthermia, induced, drug therapy, peritoneal neoplasms, surgical procedures, operative, poly (ADP-ribose) polymerases

## Abstract

Hyperthermic intraperitoneal chemotherapy (HIPEC) has emerged as a main comprehensive treatment of epithelial ovarian cancer (EOC). Despite much criticism to this approach, HIPEC has shown cost-effective benefits in both progression-free survival and overall survival for high tumor burden with no important impairment on patient-reported quality of life. On the other hand, the landscape of EOC treatment is changing rapidly and poly (ADP-ribose) polymerase inhibitors (PARPi) currently play an important role in the management of EOC based on recent trials. At this point, an important question to be scrutinized is what to expect from up-front HIPEC in the era of amazing benefits by the PARPi. Herein, we discuss the promising role of combining PARPi and HIPEC in the management of advanced EOC.

## Clinical commentary

Hyperthermic intraperitoneal chemotherapy (HIPEC) has emerged as a main comprehensive treatment of epithelial ovarian cancer (EOC) since this gynecologic malignancy spread early as a peritoneal-borne disease. Despite much criticism to this approach^[1-3]^, HIPEC proved beneficial in both progression-free survival (PFS) and overall survival (OS) for high tumor burden EOC at the time of interval debulking surgery in the OV-HIPEC trial by van Driel *et al*.^[4]^ This Randomized Clinical Trial (RCT) also showed no important differences in toxicity or patient-reported outcomes between the study groups^[5]^, and demonstrated the cost-effective benefit of HIPEC^[6]^. Based on these data, HIPEC is now fully reimbursed by Dutch health care insurance and it was included as a treatment option in the most recent National Comprehensive Cancer Network guidelines.

This multimodal approach proved to be an effective curative treatment or a salvage therapy for patients suffering from peritoneal surface malignancies^[7]^ and is currently the standard of care for appendiceal epithelial neoplasms and pseudomyxoma peritonei syndrome as well as diffuse malignant peritoneal mesothelioma^[8]^. The rationale of HIPEC is based on the direct cytotoxicity of hyperthermia for malignant cells, the enhancement of cytotoxicity of anticancer drugs, and the pharmacokinetic advantages of the intraperitoneal route for chemotherapy. Some studies also revealed that hyperthermia can reduce the mechanisms of cellular resistance to platins^[9-11]^ and induce an efficient anticancer immune response via exposure of cell surface heat shock proteins^[12,13]^. Furthermore, this technique is delivered intraoperatively, avoiding the need for implantation of peritoneal access devices and thereby reducing catheter-related morbidity and negating the issues of tolerance.

On the other hand, the landscape of EOC treatment is changing rapidly and poly (ADP-ribose) polymerase inhibitors (PARPi) currently play an important role in the management of EOC. PARPi drugs moved into the first-line treatment based on the recent SOLO-1^[14]^, PRIMA^[15]^, VELIA^[16]^, and PAOLA-1 trials^[17]^, and they have previously demonstrated an important role in maintenance in recurrent platinum sensitive EOC, fallopian tube, and primary peritoneal cancer^[18]^. At this point, an important question is what to expect from HIPEC in the era of amazing benefits by the PARPi. In other words, is it worth adding HIPEC to the comprehensive management of EOC in the PARPi era?

Systemic therapy such as both neoadjuvant (NACT) and adjuvant platinum-based chemotherapy represents the backbone of treatment in EOC. The Gynecologic Oncology Group (GOG) #111 trial compared cisplatin and cyclophosphamide with cisplatin and paclitaxel, establishing the current standard of combination chemotherapy with a platinum and a taxane^[19]^. A subsequent GOG trial demonstrated the therapeutic equivalence of cisplatin and carboplatin, but with a better toxicity profile with fewer gastrointestinal, renal, metabolic, and leukopenic events favoring carboplatin^[20]^. The doublet of carboplatin and paclitaxel was then elected as the main regimen of systemic therapy. Four main GOG trials (i.e., 104, 114, 172, and 252) also explored the survival advantage of platinum-based IP chemotherapies for EOC, establishing these regimens as preferred for the IP route of chemotherapy^[21]^. Accordingly, regimens such as cisplatin-alone, cisplatin plus doxorubicin, and carboplatin-alone have also been preferred for HIPEC in EOC^[22]^.

Platinum-based agents are one of the most widely used categories of interstrand crosslinking (ICL) agents and have been used in clinical practice for more than 50 years. These ICL drugs from the larger family of alkylating agents are able to block the separation of DNA strands, interfering with the fundamental processes of DNA transcription and replication. In these settings, platinum-based agents trigger the DNA damage response (DDR), creating a favorable “molecular landscape” that potentiates the activities of DDR-targeting agents to maintain the platinum responses or possibly induce synthetic lethality^[23]^. Nowadays, the most commonly used DDR-targeting agents are the PARPi.

A review of this issue leads to interesting findings from a clinical point of view. Some experts continue to strongly support the use of IV/IP therapy for optimally debulked patients, particularly those with no gross residual disease and those with BRCA mutations or other manifestations of homologous repair (HR) deficiency^[21]^. Accordingly, translational analysis of GOG #172 trial^[24]^ and a comparative study^[25]^ have demonstrated an improvement in PFS and OS in BRCA carriers compared to their peers treated with IV chemotherapy alone. Similar findings were also reported favoring HIPEC in BRCA mutation carriers^[26,27]^, the same main targeted patients of PARPi drugs. From a surgical scope, even in experienced centers, early tumor regrowth has been observed after complete cytoreduction in 22%-28% of patients at the time of postoperative radiological assessment before initiation of adjuvant chemotherapy^[28]^. This clearly demonstrates the need of earlier initiation of adjuvant treatments after debulking surgery in order to increase patient’s survival with EOC, and HIPEC may represent a promising way to target this issue. Additionally, 60% of patients with advanced serous papillary peritoneal carcinoma treated with NACT followed by total parietal peritonectomy plus HIPEC showed microscopic disease when no disease was macroscopically evident at surgical exploration^[29]^. For advanced EOC in general, previous studies have reported 36% of patients with disease on pathological assessment with a normal looking visual assessment by surgeons^[30]^, whereas a high incidence of pathological residual disease has been observed even after a median of five cycles of neoadjuvant chemotherapy was used before interval debulking surgery^[31]^. In these settings, HIPEC also proved to have a role for EOC based on data of the OV-HIPEC trial by van Driel *et al*.^[4]^, especially due to the higher risk of developing platinum resistance after NACT^[32]^.

The mechanisms associated with resistance to platinum-based agents mainly involve altered cellular accumulation and cytosolic inactivation/metabolism of the agent, and altered DNA repair^[23]^. Similarly, data on post-progression treatment after olaparib as maintenance therapy in patients with BRCA mutated recurrent platinum sensitive EOC seem to suggest cross resistance with chemotherapy in real world setting^[33]^. Of note, previous studies have demonstrated similar survival in both platinum-sensitive and platinum-resistant disease for patients who underwent HIPEC^[9,11]^, showing that HIPEC can reverse or circumvent the resistance to platinum-based chemotherapy and may be used in addition to PARPi, including for non-BRCA carries. Herein, inhibition of HR mechanisms^[34]^, activation of heat-shock proteins that are able to modify multiple cellular functions^[10,12,13]^, and epigenetic alterations^[35]^ are the main mechanisms involved. Pre-clinical work has also shown that mild hyperthermia (i.e., 41 °C-42.5 °C) induces degradation of BRCA2 and inhibits homologous recombination, suggesting HIPEC could be used to sensitize innately HR-proficient tumor cells to PARPi^[34]^. According to Schaaf *et al*.^[36]^, hyperthermia by HIPEC delays the repair of DNA damage caused by cisplatin or doxorubicin, acting upstream of different repair pathways to block histone polyADP-ribosylation (PARylation), and it also blocks this histone modification as efficiently as pharmacologic PARPi, producing comparable delay in DNA repair, induction of double-strand breaks, and cell cytotoxicity after chemotherapy^[36]^. Filling the gap of how to combine HIPEC and PARPi and which is the best population to benefit from both treatments is still warranted.

HIPEC has been applied at different time points of treatment of advanced EOC, namely up-front, interval, and recurrent settings^[22]^. However, the use of HIPEC at the time of interval debulking surgery is the most promising because is based on a higher level of evidence^[4]^ and also may reverse the platinum-resistance induced by NACT^[32]^, and because of the need of treatment intensification for compensating the poorer prognosis of many patients with high tumor burden that require NACT as a primary approach. In these settings, translational studies could confirm the potential synergistic effect of HIPEC and PARPi exploring some improvements by inhibiting PARP1-dependent DNA replication arrest, as previously reported by Schaaf *et al*.^[36]^ For clinical trials, we suggest exploring concentration-based HIPEC protocols instead of body surface area-based regimens of chemotherapy such as a doublet of cisplatin (42 mg/L of perfusate) plus doxorubicin (15 mg/L of perfusate) using the closed-abdomen technique with increased intra-abdominal pressure, as previously discussed^[37]^. This regimen could be applied for just 60 min to reduce the morbidity and, consequently, the costs related to HIPEC itself^[38]^, and it could be initially explored in a small randomized proof of concept Phase 2 superiority trial^[39]^ and/or in comparison to the regimen by van Driel *et al*.^[4]^ as a Simon’s randomized Phase 2 design to select the most promising regimen in terms of comprehensive outcomes and overall survival^[40]^ for further Phase 3 studies associating HIPEC and PARPi.

In conclusion, HIPEC remains an important treatment option for advanced EOC in the era of PARPi. To improve outcomes, targeted patients should be those with immunohistochemistry proven pathogenetic type I tumors and those with manifestation of HR deficiency such as BRCA carriers^[26,27,41]^. Additionally, a promising role for combining PARPi and HIPEC in non-BRCA carriers as well in post-PARPi recurrences should be prospectively explored.

## Declarations

### Copyright

© The Author(s) 2020.
